# Meeting Review: Plant Bioinformatics at the NSF and NPGI (PAMGX Satellite) Meetings

**DOI:** 10.1002/cfg.158

**Published:** 2002-04

**Authors:** Richard Bruskiewich

**Affiliations:** International Rice Research Institute (IRRI) Metro Manila DAPO 7777 Philippines

## Abstract

This brief meeting review summarizes the recommendations of NSF and NPGI funded bioinformaticians concerning the future requirements for plant bioinformatics systems and databases.


**Comparative and Functional Genomics**
*Comp Funct Genom* 2002; **3**: 176.
Published online 14 March 2002 in Wiley InterScience (www.interscience.wiley.com). **DOI:** 10.1002/cfg.158

**Conference Review**

### Meeting Review: Plant Bioinformatics at the NSF and NPGI (PAMGX Satellite) Meetings

Richard Bruskiewich*
*International Rice Research Institute (IRRI), DAPO 7777, Metro Manila, The Philippines*

*Correspondence to: International Rice Research Institute (IRRI), DAPO 7777, Metro Manila, The Philippines. E-mail: r.bruskiewich@cgiar.org

Received: 1 February 2002
Accepted: 13 February 2002

### Abstract

**This brief meeting review summarizes the recommendations of NSF and NPGI funded bioinformaticians concerning the future requirements for plant bioinformatics systems and databases. Copyright © 2002 John Wiley & Sons, Ltd.**

On Friday, 11th January, 2002, the National Science Foundation (NSF) and the National Plant Genomics Initiative (NPGI) hosted meetings concerning diverse scientific activities within their respective programs. Included in both meetings were specific sessions on plant bioinformatics, including reports on the current implementations of plant databases and considerations for future development. Several leading bioinformaticians of these databases were in attendance.

These meetings follow up on an earlier NSF-sponsored meeting of plant bioinformaticians last September 11th, 2001 held at TIGR. The summary recommendations coming out of the earlier meeting were as follows:

- All databases must have capabilities that will allow the broadest access by the community
- The community should begin developing a standard format for data exchange
- Metadata (UML or XML defined) must be defined for complementary human and machine parsing
- Data release policies and standard operating procedures should be posted at data sites
- Agencies need to fund organism independent software, data archives and annotation services
- Agencies should support open-source, including the development of adequate documentation
- Complete (raw) data must be archived including EST trace files, raw map data and data files for integrated physical and genetic maps

The NSF meeting white papers on bioinformatics and databases along with the final report of the September meeting are available on the WWW at **http://plantgenome.sdsc.edu/**.

Particular emphasis was made concerning the development of more extensive open community protocols for data exchange and archival of complete data. Following the spirit of the 'Minimal Information About a Microarray Experiment' (1) efforts of the Microarray Gene Expression Database (MGED) consortium (**http://www.mged.org/**), the group proposes to develop 'Minimal Information About a Functional Genomics Experiment' (MIAFGE), extending the concept of MIAME to ALL functional genomics experimental data. As a particular example of the necessity to properly archive original ('raw') data, it was noted that the underlying raw segregation data for individual mapping populations of many historical comparative mapping efforts are no longer available. Proper community archives for such data need to be established for current and future comparative mapping efforts.

**Disclaimer**: The writer was a general participant in the NSF Awardees' and NPGI satellite meetings at the Plant, Animal and Microbe Genome X (PAMG-X) conference, but was not part of the original bioinformatics review panel. This review is solely based upon the writer's third party interpretation of meeting discussions and handouts, and is merely provided as an unsolicited (and NSF/NPGI unofficial) public information service to the readers of this journal.

### References

1. Brazma A, Hingamp P, Quackenbush J, *et al.* 2001. Minimum information about a microarray experiment (MIAME) – toward standards for microarray data. *Nature Genetics* **29**: 365–371.

Copyright © 2002 John Wiley & Sons, Ltd.

## References

[PDF_00082] Brazma A., Hingamp P., Quackenbush J., Sherlock G., Spellman P., Stoeckert C., Aach J., Ansorge W., Ball C. A., Causton H. C. (2001). Minimum information about a microarray experiment (MIAME)-toward standards for microarray data.. Nat Genet.

